# Retaining early career registered nurses: a case study

**DOI:** 10.1186/s12912-016-0177-z

**Published:** 2016-10-10

**Authors:** Jane Mills, Jennifer Chamberlain-Salaun, Helena Harrison, Karen Yates, Andrea O’Shea

**Affiliations:** 1 Centre for Nursing and Midwifery Research, College of Healthcare Services, James Cook University, PO Box 6811, Cairns, Queensland 4870 Australia; 2 School of Health & Biomedical Sciences, RMIT University, Plenty Road, Bundoora, Victoria 3083 Australia; 3 Queensland Health, Cairns, Australia

**Keywords:** Early career, Registered nurse, Retention, Support

## Abstract

**Background:**

A core objective of the Australian health system is to provide high quality, safe health care that meets the needs of all Australians. To achieve this, an adequate and effective workforce must support the delivery of care. With rapidly changing health care systems and consumer demographics, demand for care is increasing and retention of sufficient numbers of skilled staff is now a critical priority to meet current and future health care demands. Nurses are the largest cohort of professionals within the health workforce. Reducing the rates at which nurses leave the profession and supporting nurses to practice in their profession longer will have beneficial implications for the sustainability of a nursing workforce and, ultimately, to patient outcomes. The aim of the study was to describe and explain early career registered nurses’ (ECRNs) experiences and support requirements during the first five years of practice for the purposes of identifying strategies that would support greater retention of ECRNs.

**Methods:**

A single case study design focused on early career registered nurses (ECRNs) working in a hospital and health service in northern Australia. The research team adopted Djukic et al’s definition of ECRNs as “RNs who have practiced for less than 5 years”. Data was collected via three individual interviews and two focus groups. Thirty-five ECRNs participated in the study.

**Results:**

Qualitative analysis of data generated during interviews and focus groups, identified the key themes of *receiving career advice* and *choice or no choice*. Analysis of study data in the context of the broader literature resulted in the researchers identifying six areas of focus for ECRN retention: 1) well-planned, supported and structured transition periods; 2) consideration of rotation through different areas with a six month minimum for skills development; 3) empowering decision making; 4) placement opportunities and choice in decisions of where to work; 5) career advice and support that considers ECRNs’ personalities and skills; and 6) encouragement to reflect on career choices.

**Conclusions:**

Reducing turnover and improving retention relies on understanding the factors that influence nurses’ decisions to leave or remain within an organisation and the profession. Ensuring nurses in the current workforce remain engaged and productive, rather than leave the profession, is reliant on addressing factors that cause attrition and implementing strategies that strengthen retention rates and workforce sustainability.

## Background

The importance of nursing to the national infrastructure of health care in Australia is well recognised [1–3]. As the largest cohort of professionals within the health care workforce, registered nurses [RNs] have an essential role in the delivery of health care [1, 3–5]. Predicted workforce shortages concomitant with the retirement of an aging nursing workforce and increasing rates of nurse turnover, particularly early career registered nurses [ECRNs], has drawn attention to issues influencing nurse turnover and retention [2, 6–8]. In the context of this study, the authors have adopted Djukic et al’s definition of ECRNs as “RNs who have practiced for less than 5 years” [9].

HWA [2, 3] reports that the probability of permanent exits from the Australian nursing workforce is highest among nurses aged over 60 years and under 35 years of age. Some of these exits are expected and understood: nurses are ageing and retiring and younger nurses are leaving for reasons such as starting a family [2, 6]. The reasons for the high turnover of ECRNs, however, are unclear and concerning. Similarly the key to retaining ECRNs is elusive with important factors yet to be fully explored [7, 8, 10, 11]. Loss of ECRNs threatens the long-term sustainability of the health care workforce and the capacity of health systems to provide quality care. Studies relating to ECRN turnover and retention predominately focus on nurses in their first year of practice and commonly relate to challenges associated with transition from student to professional [8, 12, 13]. Little is known about ECRNs beyond their transition year.

Turnover rates of nurses reported in the literature vary and range between 5 and 60 % [8, 11, 14]. Studies of the Australian nursing workforce estimate the overall annual turnover rate to be 15.1 % per year with state variances ranging between 12 and 38 % [8, 11]. While there are no specific estimates related to ECRNs as a group, there are suggestions in the literature [8, 10, 13–15] that rates of nurse turnover worldwide in the first 12 months after graduation are between 17 and 50 %. Nurse turnover is complex and high turnover has economic, nurse and patient care consequences [8, 11, 14, 16–19].

The financial implications of nurse turnover include loss of public funds and staff replacement costs [8, 14, 18, 19]. The loss to individual and community investment in educational preparation, when a nurse leaves the profession, is estimated to be approximately AUD$150,000 [7]. Costs associated with replacing staff include employing temporary replacements, recruitment and loss of productivity [11, 14]. When vacancies exist and temporary cover is high, staffing continuity is disrupted, productivity is reduced and nurse patient ratios and skills mix are affected. These factors impact on the efficiency and quality of care and patient safety [8, 16, 17, 20]. Nurse turnover destabilises nursing teams, increases workloads, alters clinical practice and generates high levels of stress and burnout, all of which contribute to job dissatisfaction [16, 21]. High prevalence of job dissatisfaction has been linked to both nurses’ intent and nurses’ decisions to leave the profession [8, 12, 22].

Improving nurse retention reduces costs associated with turnover and improves nursing productivity and sustainability, and the collective return on investment [2, 7, 23]. Friedman et al. [24] found that implementing a support program for new graduate nurses in critical care reduced turnover and yielded a cost saving of approximately $1,367,100 annually. The added value of nurse retention to patient and health care professional satisfaction, employee engagement, organisational success and patient outcomes heightens the impetus to reduce turnover and enhance retention.

Factors that influence retention of RNs in their first year of practice have been well documented [7, 8, 16, 20]. Less is known, however, about the needs of ECRNs beyond the transition year and the factors, which influence their career intentions and retention plans. Studies exploring nursing turnover and retention give some insight into what might support new graduates. However, this group is often generationally younger, attitudinally different and seeking different goals and approaches to life and work [25–28]. Consequently, their needs and expectations may be different as they move beyond transition and embed themselves into the workforce [25, 27]. Acquiring greater understanding of ECRNs’ experiences, beyond the first year of practice, including factors that underpin their decisions to stay or leave could provide valuable insight to strategies that support them in the workplace and keep them engaged in the profession.

### Aim

The aim of the study was to describe and explain the case of ECRNs’ experiences and support requirements during the first five years of practice.

## Methods

### Design

The research team used a single case study design, which is defined as an intensive study of an individual unit of interest [29] with a focus on the developmental factors of that unit [30]. The case study in the project focused on ECRNs’ support requirements in one public hospital located within in a Hospital and Health Service (HHS) in northern Australia. As at October 2015, there were 1032 RNs working in the HHS, which covers an area of 141,000 km^2^.

### Sample

The HHS Human Resource department provided the research team with a list of ECRNs. Letters of invitation to participate in interviews and focus groups were mailed to 58 ECRNs and were followed up by an email reminder. Purposive sampling resulted in a total of 35 ECRNs participating in the study.

### Data generation

Data was generated between December 2014 and May 2015 via three individual participant interviews (PI) conducted via telephone and two focus groups (FG) of 10 and 22 participants respectively. Interviews and focus groups followed a semi-structured format guided by the following topics:

- Factors impacting the transition from student to RN
- Factors impacting professional development of nurses during the first five years of practice
- Balancing work-life responsibilities
- Opportunities for career advancement
- Key points/triggers for career decisions
- Useful support strategies in the first five years of practice

### Analysis

Interviews and focus group discussions were audiotaped and professionally transcribed. Microsoft® Word version transcriptions were imported into NVivo for MAC data management software, version 10.2.1. Transcripts were open coded and the grounded theory method of constant comparative analysis [31] was used to group codes into themes corresponding to areas of support as identified by study participants.

## Results

The period of transition from student to registered nurse is a period in which nurses are finding their place in the healthcare environment. Study findings suggest that finding one’s place or “niche” within the hospital is an important factor in ECRNs’ professional development. Finding one’s niche benefits from and is hindered by *receiving career advice* and having *choice or no choice* in their career development.

### Receiving career advice

ECRNs want to be able to establish themselves as nurses and find their ‘niche’ in the hospital but often do not have the confidence to direct their own career path. Receiving career advice is both confusing and beneficial for ECRNs depending on who is offering the advice. Well-intentioned, unsolicited advice from more experienced nurses can lead to ECRNs feeling confused and can also influence their decisions about which units to apply to work in within the hospital. Four participants in one focus group provided the following comments:

*I had about four or five different nurses telling me I should go to another ward, or I should try this hospital or that hospital. *(FG1)

*You come out as a grad nurse you want to be able to establish yourself in - a certain area. But when people saying to you, “No-no, go and work in this ward first so you can get your skills up, and then apply for that.” *(FG1)

*I had so many nurses telling me I shouldn’t do theatre right away. *(FG1)

*So I find on some wards I’ve been on you have some nurses that reckon you shouldn’t be here, “You should try out that one. That one’s much nicer. You should go there,” or “Stay at this one, don’t leave.” […] every nurse has their own opinion on where you should be going or where you should stay. *(FG1)

Receiving advice can also have positive implications. Participants in one of the focus groups spoke about a member of the hospital human resources team providing them with objective career advice and support:

*She was absolutely fantastic […]. She was like a career advisor. […] I was going to quit nursing. She’s the one that told me, “There’s more areas than [the one you’re working in”.* (FG1)

*She’d give you her [work] number and say, “Call me whenever you need to” and she was the one that told me I should stick with theatre. If I wanted to do theatre, stick with it. […] She was really good. We need someone like her. […] you had that support and you knew you could call somebody if you really needed to.* (FG1)

When focus group participants were asked whether they thought it would be beneficial having a career advisor to assist them in identifying which unit may suit their personality and their skills, there was a resounding yes. This prompted a participant to share her experiences of attending a university careers day and completing an online questionnaire about suitable careers. Based on the participant’s questionnaire responses, midwifery, nursing and health care were considered suitable career options, which triggered the participant to change her university degree to nursing. The participant added, “If we had something like that here [in the hospital] it would help us decide, well, where do we fit? For those that haven’t found it [their niche].” (FG2)

### Choice or no choice

In relation to employment opportunities within the HHS, ECRNs highlighted that gaining employment in their preferred hospital unit is not always a matter of choice. One participant’s comment sums up the situation for new graduates, “you’re at the mercy of where you end up on your first position whether it’s where you chose or not and what you are aware of as well” (FG2). Some study participants do not feel that they had “ever made a decision” (PI3) in relation to their career, as the following participant explained:

*I applied for [ward x] when I first finished my grad year, and didn’t get it. The only place I could get a job was on [ward y], which is where I did not want to be. But I went and did that anyway, and I really, really enjoyed it. So I nearly spent two years there, but burnt out on that ward, and then got offered a job on nursing support, where I travelled all around the hospital. […] So I don’t feel like I’ve ever really made a decision in my career. *(PI3)

Having no choice as to which unit ECRNs are employed in, can also have positive consequences:

*I’m just first-year, and I applied for [Unit a] and I didn’t get it, and then I got [Unit b] and I don’t regret that at all. It wasn’t really a choice, even though it was an offer, but I love it. So I’m happy where I am. Yeah. It was kind of out of my control. *(FG1)

*Initially I didn’t really want to specialize straightaway. I wanted to just maybe – general wards. Get some skills up and then progress through. But once it was offered to me [position in specialized area], I just leaped at the chance of getting a job really, and I went from there. *(PI1)

ECRNs, in both interviews and focus groups, identified planned rotations as a strategy that could address issues of *choice or no choice* as highlighted in participants’ comments above. There was overwhelming support among participants for the opportunity and the value of trying out different units within the hospital as the following comments demonstrate:

*I haven’t found my niche here, and it would be nice to just go and have a taste of this, and a taste of that, just for the experience. *(FG1)

*If that was available, I would definitely have done that because then I would have got a taste and a feel of the areas and a little bit more – I suppose keeping your skills and gaining skills. *(FG1)

*I wouldn’t mind getting a taste of different wards and seeing what I would actually like. *(FG2)

Study participants are aware of rotation opportunities and/or graduate programs offered by other HHS. The following participants’ comments highlight, what they perceive, as the value of gaining experience in a range of units:

*I’ve been told by lots of different nurses […] that this hospital is the only one that doesn’t do the grad program like other hospitals so, where you spend three months in this unit, three months in that, three months – and you get a whole bunch of skills and you’re supported and you still study during that time so you feel a lot more prepared. *(FG1)

*The other hospitals seem to have at least three month or four month rotations and after a year then you can put your preference to where you want to spend your next 12 months and that’s giving you a little bit of a taste of what you can do and what you can’t do, what you like and what you don’t like. I think it’s a better way to approach. […] you get a sense of what other nursing styles are about. *(FG2)

*[the hospital] doesn’t do it too well with their new grad program where they don’t do a rotation through [… like some of the Brisbane hospitals do. I had some peers that went through nursing that got really good – from their experience, got really good rotations. So did four different wards in the first year whereas it’s not offered as freely in [regional city]. It’s quite hard to come by actually. I think having access to multi-areas to figure out what exactly you want to do - you get a quick glimpse of it when you’re doing the uni [university] training but I’ don’t really think it’s enough to decide where you want to go and progress on in just the three or six weeks that you do your clinical practice in. *(PI1)

The same participant added that they believe “six months is probably a minimum if you did a rotation through different areas” (PI1).

Working as a student in nursing is also considered a valuable strategy for familiarising ECRNs with the different units within a hospital and for solidifying, more immediately, what is being studied at university. One participant explained her experience as follows:

*[it] helped tremendously. […] it was good to just shadow them [RNs] while I was at uni [university] and then just the things I was studying were coming up on my shift and I was like, “Oh, I just learnt about that in a lecture yesterday”.* (FG2)

Some ECNs also found that undertaking their final clinical placement as student nurses in the unit that they wanted to work in enabled them to build relationships. Having previously established collegial relationships facilitates the transition from student to ECRN and enables ECRNs to find their niche more quickly.

*I was happy enough to have two placements in theatre before I started and you build rapport with people. So when I did start, it was like […] you just slid back into where you were, which was a huge part of my transition – there were heaps of familiar faces and it was just easier to slide in and just get on with it. *(FG2)

## Discussion

This study sought to describe the experiences of ECRNs to better understand their support requirements in the first five years of practice for the purposes of aiding retention. Participants identified a number of factors that either enhanced or impeded their transition from student to registered nurse. The ECRNs in this study identified that they often found it difficult to identify where their niche in nursing was and received career advice that was often unsolicited and conflicting. Some colleagues advised ECRNs to remain in one area and avoid specialisation until skills are consolidated, while others encouraged ECRNs to specialise early in their desired area. Stokowski [32] noted that in the past, new graduate nurses were discouraged from specialising in the belief that they first needed to develop basic skills. Participants identified that a reliable source of professional career advice would have helped overcome the confusion. The provision of professional career advice has previously been identified as an unmet need for new graduates and has been noted to impact on retention [33].

When applying for positions in the HHS, participants in this study were able to provide preferences for the clinical area in which they wanted to work but often these preferences could not be met. This meant that some ECRNs worked in areas they had not considered before with varied impact on their feelings of job satisfaction and potential to leave. While some participants identified that although they had not considered the clinical area in which they were employed, they nonetheless found it satisfying; others, however, felt trapped in what they perceived as an undesirable clinical area. Job availability for ECRNs is a known contributor to accepting a position not in an area of choice [34]. In the absence of choice factors that become important to ECRNs include: how they are treated by other staff members, teamwork and learning opportunities [34]. The option to move to another clinical area or the security of planned and structured rotations in the first year can also help overcome ECRNs’ dissatisfaction with their initial placement [10].

This existing evidence was supported by findings from this study, which identified the potential of rotations as a strategy to enhance job satisfaction. Many new graduate programs are structured around three to four rotations in the first year [28] but this can have both positive and negative implications for ECRNs. When their colleagues know they are on a short appointment new registered nurses may not be supported to feel that they belong in a particular team. Short appointments and frequent rotations may also impact on the development of an ECRN’s clinical skills and confidence [35, 36]. Participants in this study supported the idea of rotations as a way to overcome a lack of choice in an ECRN’s first placement but acknowledged that six-month rotations were required to allow for the consolidation and development of knowledge and skills.

A number of participants identified that employment as a student nurse helped them to both identify areas of clinical preference but also aided the transition to working as a registered nurse. Benefits to working as a student nurse or assistant in nursing are identified in the literature. These benefits include earlier development of confidence and critical thinking skills, greater familiarity with the practice environment and the ‘real world’ of nursing, and enhanced assimilation into the clinical environment [37, 38].

Recommendations to improve ECRN retention arising from the study findings are to ensure:

1. Well-planned, supported and structured transition periods
2. Graduate nurse rotation through two different clinical areas with a six month minimum for consolidation and development of knowledge and skills
3. Structured provision of placement opportunities during the first five years of a registered nurses’ career
4. Career advice and support that accounts for ECRN’s personality, goals, experience and skills
5. Participation in career development opportunities that assist ECRNs to make strategic decisions about their career development
6. Encouragement to reflect on and plan career choices

## Conclusion

Current evidence about the cost and impact of registered nurse turnover indicates an urgent need to identify strategies to improve retention. Longer-term high registered nurse turnover rates will impact negatively on patient outcomes, the cost of providing healthcare services and the human capital available to lead the provision of these services in the future. Reducing turnover and improving retention relies on understanding the factors that influence nurses’ decisions to leave or remain within an organisation and the profession. Ensuring nurses in the current workforce remain engaged and productive relies on addressing factors that cause attrition, and implementing strategies that strengthen retention rates and workforce sustainability. The added value resulting from these strategies will provide a platform for organisational success as well as the long-term sustainability of the nursing workforce.

### Limitations

Findings are limited to ECRNs from one hospital and health service located in one Australian State. Findings, therefore, may not be representative of ECRNs in other locations or facilities.

## Abbreviations

ECRN: Early career registered nurse
HHS: Hospital and health service
RN: Registered nurse

## References

[1] Australian Institute of Health and Welfare (2014). Australia’s health 2014. Australia’s health series no.13. (Cat. No. AUS 178).

[2] Health Workforce Australia (2012). Health workforce 2025-doctors, nurses and midwives– Volume1.

[3] Health Workforce Australia (2014). The Australia’s future health workforce – nurses overview report.

[4] Australian Institute of Health and Welfare (2012). Nursing and midwifery workforce 2011. National health workforce series. no. 2. (Cat. No. HWL 48).

[5] Leadership for the Sustainability of the Health System: Part 1 - A Literature Review [http://www.springboard.health.nsw.gov.au/content/uploads/2014/07/leadership-for-sustainability-of-healthsector-literature-review-012012.pdf] Accessed 7 Oct 2016.

[6] Health Workforce Australia (2012). Patterns and determinants of medical and nursing workforce exits– final report.

[7] Australian Nursing Federation (2013). Submission to the health workforce Australia consultation paper on nursing workforce retention and productivity.

[8] Hayes L, O’Brien-Pallas L, Duffield C, Shamian J, Buchan J, Hughes F, Laschinger H, North N (2012). Nurse turnover: a literature review – an update. Int J Nurs Stud.

[9] Djukic M, Kovner C, Brewer C, Fatehi F, Seltzer J (2013). A multi-state assessment of employer-sponsored quality improvement education for early-career registered nurses. J Contin Educ Nurs.

[10] Cubit K, Ryan B (2011). Tailoring a graduate nurse program to meet the needs of our next generation nurses. Nurse Educ Today.

[11] Roche M, Duffield C, Homer C, Buchan J, Dimitrelis S (2014). The rate and cost of nurse turnover in Australia. Collegian.

[12] Laschinger H (2012). Job and career satisfaction and turnover intentions of newly graduated nurses. J Nurs Manag.

[13] Salt J, Cummings G, Profetto-McGrath J (2008). Increasing retention of new graduate nurses. J Nurs Adm.

[14] Duffield C, Roche M, Homer C, Buchan J, Dimitrelis S: A comparative review of nurse turnover rates and costs across countries. J Adv Nurs. 2014a; 70(12):2703–2712. 10.1111/jan.12483 25052582

[15] Healy M, Howe V (2012). Study of Victorian early graduate programs for nurses and midwives.

[16] Dawson A, Stasa H, Roche M, Homer C, Duffield C (2014). Nursing churn and turnover in Australian hospitals: nurses perceptions and suggestions for supportive strategies. BMC Nurs.

[17] Aiken L, Sloane D, Bruyneel L, Van Den Heede K, Griffiths P, Busse R, Diomidous M, Kinnunen J, Kozka M, Lesaffre E (2014). Nurse staffing and education and hospital mortality in nine European countries: a retrospective observational study. Lancet.

[18] Buerhaus P, Staiger D, Auerbach D (2009). The future of the nursing workforce in the United States: data, trends and implications.

[19] Li Y, Jones C (2013). A literature review of nursing turnover costs. J Nurs Manag.

[20] North N, Leung W, Ashton T, Rasmussen E, Hughes F, Finlayson M (2013). Nurse turnover in New Zealand: costs and relationships with staffing practises and patient outcomes. J Nurs Manag.

[21] Baumann A (2010). The impact of turnover and the benefit of stability in the nursing workforce.

[22] Cowin L (2002). The effects of nurses’ job satisfaction on retention: an Australian perspective. J Nurs Adm.

[23] Health Workforce Australia (2014). Nursing workforce sustainability, improving nurse retention and productivity.

[24] Friedman M, Cooper A, Click E, Fitzpatrick J (2011). Specialized new graduate RN critical care orientation: retention and financial impact. Nurs Econ.

[25] Brewer C, Kovner C, Greene W, Tukov-Shuser M, Djukic M (2012). Predictors of actual turnover in a national sample of newly licensed registered nurses employed in hospitals. J Adv Nurs.

[26] Beecroft P, Dorey F, Wenten M (2008). Turnover intention in new graduate nurses: a multivariate analysis. J Adv Nurs.

[27] Riegel E (2013). Orienting a new generation of nurses: expectations of the millennial new graduate. Open J Nurs.

[28] Phillips C, Esterman A, Smith C, Kenny A (2013). Predictors of successful transition to registered nurse. J Adv Nurs.

[29] Stake R (1995). The art of case study research.

[30] Flyvbjerg B, Denzin NK, Lincoln YS (2011). Case study. Handbook of qualitative research.

[31] Birks M, Mills J (2015). Grounded theory: a practical guide.

[32] Stokowski L: Does your personality match your nursing speciality? Medscape http://www.medscape.com/viewarticle/838732*.* Accessed 1 Sept 2015.

[33] Robinson S, Murrells T, Clinton M (2006). Highly qualified and highly ambitious: implications for workforce retention of realising the career expectations of graduate nurses in England. Hum Resour Manag J.

[34] Hewitt P, Lackey S, Letvak S (2013). New graduate survey: factors that influence new nurses’ selection of first clinical position. Clin Nurse Spec.

[35] Evans J, Boxer E, Sanber S (2008). The strengths and weaknesses of transitional support programs for newly registered nurses. Aust J Adv Nurs.

[36] Henderson A, Ossenberg C, Tyler S (2015). ‘What matters to graduates’: an evaluation of a structured clinical support program for newly graduated nurses. Nurse Educ Pract.

[37] Romyn D, Linton N, Giblin C, Hendrickson B, Houger Limacher L, Murray C, Nordstrom P, Thauberger G, Vosburgh D, Vye-Rogers L et al. Successful transition of the new graduate nurse. Int J Nurs Educ Scholarsh. 2009;6(1):Article 34. doi:10.2202/1548-923X.1802. 10.2202/1548-923X.1802 19883374

[38] Missen K, McKenna L, Beauchamp A (2014). Satisfaction of newly graduated nurses enrolled in transition‐to‐practice programmes in their first year of employment: a systematic review. J Adv Nurs.

